# RETRACTION: Antibiofilm and Antimicrobial Activity of Lactobacillus Cell Free Supernatant Against *Pseudomonas aeruginosa* Isolated from Burn Wounds

**DOI:** 10.1111/iwj.70252

**Published:** 2025-02-25

## Abstract

**RETRACTION:** AsadzadeganR. , HaratianN. , SadeghiM. , MaroufizadehS. , MobayenM. , SaraeiH. S. E. , and Hasannejad‐BibalanM. , “Antibiofilm and Antimicrobial Activity of Lactobacillus Cell Free Supernatant Against Pseudomonas aeruginosa Isolated from Burn Wounds,” International Wound Journal 20, no. 10 (2023): 4112–4121, 10.1111/iwj.14305.37455022 PMC10681627

The above article, published online on 16 July 2023, in Wiley Online Library (http://onlinelibrary.wiley.com/), has been retracted by agreement between the journal Editor in Chief, Professor Keith Harding; and John Wiley & Sons Ltd. Following an investigation by the publisher, all parties have concluded that this article was accepted solely on the basis of a compromised peer review process. In addition, the article does not include any information about the informed consent procedure for the study. The editors have therefore decided to retract the article. The authors disagree with the retraction. The authors have further stated that the data contained in this article is not compromised or inaccurate and that they reserve the right to submit their article to another journal, including reference to this retracted article for full transparency.

**RETRACTION:** R. Asadzadegan , N. Haratian , M. Sadeghi , S. Maroufizadeh , M. Mobayen , H. S. E. Saraei , and M. Hasannejad‐Bibalan , “Antibiofilm and Antimicrobial Activity of Lactobacillus Cell Free Supernatant Against Pseudomonas aeruginosa Isolated from Burn Wounds,” International Wound Journal 20, no. 10 (2023): 4112–4121, 10.1111/iwj.14305.37455022 PMC10681627

The above article, published online on 16 July 2023, in Wiley Online Library (http://onlinelibrary.wiley.com/), has been retracted by agreement between the journal Editor in Chief, Professor Keith Harding; and John Wiley & Sons Ltd. Following an investigation by the publisher, all parties have concluded that this article was accepted solely on the basis of a compromised peer review process. In addition, the article does not include any information about the informed consent procedure for the study. The editors have therefore decided to retract the article. The authors disagree with the retraction. The authors have further stated that the data contained in this article is not compromised or inaccurate and that they reserve the right to submit their article to another journal, including reference to this retracted article for full transparency.

